# 

**Published:** 1900-09-01

**Authors:** 


**CADBURY'S COCOA** ABSOLUTELY PURE.

**CADBURY'S COCOA** NO DRUGS OR ALKALIES USED.

### THE HOSPITAL

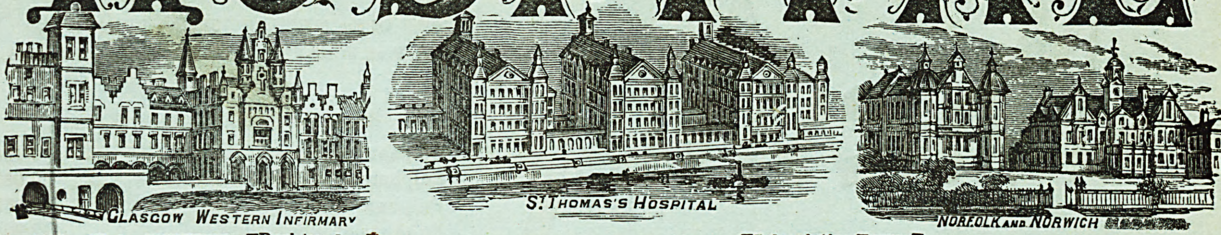

No. 727. Vol. XXVIII. [Registered as a Newspaper.] SATURDAY, SEPTEMBER 1, 1900. [Subscription Terms, see p. 364.] **PRICE TWOPENCE.**

